# Relatability as a Racialised Construct in Corporate Graduate Recruitment: Revealing a Hidden Mechanism of Labour Market Exclusion for Black African Youth in South Africa

**DOI:** 10.1111/1468-4446.70128

**Published:** 2026-05-20

**Authors:** Sifiso Mthembu, Kurt April, Cihat Erbil, Mustafa F. Özbilgin

**Affiliations:** ^1^ University of Cape Town Cape Town South Africa; ^2^ Ankara Haci Bayram Veli University Ankara Turkey; ^3^ Brunel University of London Uxbridge UK

**Keywords:** critical race theory, employer decision‐making, labour market inequality, racialised employability, relatability, social closure, youth unemployment

## Abstract

In corporate graduate recruitment worldwide, candidates are often assessed not only on competence but on whether they are deemed relatable. This study theorises relatability as a racialised cultural–affective filter that covertly sustains inequality. Drawing on qualitative interviews, we identify five interlinked processes of self‐presentation, confidence, bias, choice, and affinity, through which whiteness operates as a normative anchor in hiring. We extend theories of social closure into aesthetic and affective domains, conceptualising relatability as a meso‐level mechanism linking micro‐interactional judgements to macro‐level racial hierarchies. This framework offers a transferable analytic lens for understanding how cultural capital is operationalised in exclusionary ways across contexts, even in formally and supposedly deracialised systems. The findings call for demand‐side reforms that reconfigure organisational norms, broaden definitions of professionalism, and reduce reliance on cultural familiarity as a proxy for merit. Relatability operates as an institutionalised mechanism through which classed and racialised dispositions are recognised and reproduced, even within racially diverse hiring structures.

## 1 Introduction

In a world where trust increasingly resides in perceived similarity, the Edelman Trust Barometer (Edelman 2024) reports that 63% of people say they trust ‘people like themselves’ more than CEOs or government leaders. This preference for proximity over positional authority underscores how interpersonal affinity functions as a powerful lens through which competence and credibility are judged. Within South Africa's labour market, such affinity‐based evaluations do not simply shape interpersonal impressions but can harden into structural filters that determine access to employment. Employment crises are hardly confined to the Global South, yet the magnitude and tenacity of youth unemployment in post‐apartheid South Africa remain distinctive. Recent labour‐force statistics record an overall jobless rate of 32.9% in the first quarter of 2025 (Statistics South Africa 2025a), soaring to 62.4% among 15–24‐year‐olds in Q1 2025 (Trading Economics 2025) and 46.1% for the broader youth cohort aged 15–34 (Statistics South Africa 2025a). These headline figures conceal a still starker reality: Black African young people persistently languish at the back of the hiring queue, with unemployment rates of 36.9% among Black South Africans compared to only 7.9% among white South Africans in Q2 2024 (Statistics South Africa 2024), even when qualifications, school quality and geographic location are held constant (Kingdon and Knight 2004; Baldry 2016). Over the past decade, youth unemployment has deteriorated markedly, climbing from 36.9% in Q1 2015 to 46.1% in Q1 2025, a 9.2% point increase that underscores worsening prospects for millions (Statistics South Africa 2025b). Three decades after the formal demise of apartheid, racial hierarchies continue to orchestrate entrance to paid work (Branson et al. 2024).

These patterns extend beyond labour market dynamics and are rooted in deeply entrenched inequalities of opportunity in post‐apartheid South Africa. Educational stratification, spatial segregation, and intergenerational transmission of advantage continue to shape access to high‐quality schooling, tertiary education, and professional networks. As a result, pathways into employment are unevenly structured long before individuals enter the labour market. Relatability, a multi‐layered heuristic through which employers assess whether a candidate is perceived as fitting, familiar, and organisationally safe in contexts of uncertainty and risk, operates, we argue, as a mechanism through which these historically produced inequalities are translated into contemporary hiring decisions. While we do not claim that these dynamics apply uniformly across all segments of the labour market, we demonstrate how they operate within corporate hiring contexts where discretionary evaluation plays a central role. This study focuses specifically on corporate‐sector hiring and graduate recruitment pathways, where access to professional employment is mediated through formalised selection processes requiring matric and, in most cases, tertiary education.

Much of the extant scholarship locates the problem on the supply side, invoking macro‐economic stagnation, skill shortages and individual employability deficits. In contrast, this article explicitly centres demand‐side processes within organisations, examining how employer decision‐making actively produces labour market exclusion. Such analyses rest on human‐capital logics that presume rational, efficiency‐seeking employers. In so doing, they obscure the subtle yet potent demand‐side processes embedded in organisational norms, discretionary managerial power and racialised expectations (Bonilla‐Silva 1997; Roscigno et al. 2007). Responding to this lacuna, we conceptualise relatability as a racialised evaluative mechanism rather than as a standalone social theory. Rather than treating exclusion as a deficit located in job seekers, we analyse how evaluative criteria are constructed and applied by employers. This evaluative mechanism unfolds through five intertwined processes, namely self‐presentation, confidence, bias, choice, and affinity. Each process is cloaked in the rhetoric of neutrality yet cumulatively rewards proximity to whiteness and penalises cultural or socio‐economic divergence, particularly where middle‐class dispositions, such as accent, schooling, and institutional exposure, are unevenly distributed both across and within racial groups.

Our argument proceeds from the premise that exclusion is normalised not only through overt prejudice but also through routinised judgements that are widely perceived as commonsensical and meritocratic. We characterise these judgements as forms of unconscious incompetence, contextual apathy, limited social imaginaries, short‐termism and homophily, which together structure how employers interpret candidate suitability under conditions of informational uncertainty. To unravel these dynamics we draw upon thirteen in‐depth interviews with senior hiring professionals and labour intermediaries, complemented by two focus groups with unemployed Black African youth. Adopting a hermeneutic‐phenomenological stance, we trace the quotidian hiring conversations through which racial closure is reproduced beneath the veneer of meritocracy.

The article makes three principal contributions. First, it furnishes a conceptual vocabulary for analysing racialised exclusion by foregrounding relatability as an evaluative mechanism embedded in everyday hiring practices. Secondly, it challenges policy orthodoxies that privilege supply‐side remedies, demonstrating instead how labour market inequality is produced within corporate graduate recruitment through employer discretion and organisational routines. Thirdly, it advances methodological debate by illustrating the analytical purchase of combining hermeneutic interpretation with grounded‐theory approaches in the study of racialised decision‐making. Crucially, this article does not advance relatability as an abstract or universal theory; rather, it mobilises relatability as an analytic lens that brings together existing sociological insights on homophily, cultural capital and aesthetic labour to show how interactional hiring judgements are linked to broader structures of racial inequality in the labour market.

The manuscript unfolds as follows. We begin with a review of competing explanations for South Africa's youth unemployment and introduce the theoretical debates that inform our analysis. We then outline the research design and analytical procedures. The findings section examines how five interlinked evaluative processes combine to disadvantage Black African youth, before situating these micro‐level dynamics within broader structures of social closure. We conclude by reflecting on implications for labour market regulation, employer practice, and future scholarship.

## 2 Racial Dynamics of Youth Labour Market Exclusion in South Africa

Youth unemployment has long commanded scholarly attention, yet debates remain dominated by macroeconomic, structural and institutional perspectives. Structural analyses emphasise the entrenched segmentation of labour markets into secure primary sectors and precarious secondary ones (Doeringer and Piore 1971), a divide within which young people are disproportionately concentrated (Bell and Blanchflower 2011). Current estimates indicate that 21.7% of the global youth population is not in education, employment or training (NEET), with the rate at 27.5% for young women and 16.3% for young men, a proportion amplified by the COVID‐19 pandemic and reflecting a recovery that remains uneven across regions (International Labour Organization (ILO) 2025; Shita et al. 2025). Human capital theory frames employability as the return on investments in education and training, presuming a relatively direct translation of credentials into labour‐market access (Becker 1964). However, in South Africa, where racialised histories have structured access to schooling, internships and social capital, educational attainment alone repeatedly fails to offset inherited disadvantage; persistent skills mismatches between curricula and employer demand underline the limits of supply‐side fixes (Banerjee et al. 2025; Sarkar et al. 2019). Institutional perspectives remind us that policy regimes and organisational infrastructures shape opportunity structures (May and Jochim 2013), yet precisely because they privilege formal barriers, they tend to leave routine, informal judgements, the everyday gatekeeping of entry‐level recruitment, largely unexamined.

South African scholarship on inequality of opportunity has consistently shown that access to economic participation is mediated by historically structured differences in schooling quality, linguistic capital, and social networks (e.g., Fataar 2018; Seekings and Nattrass 2005). This body of work is further enriched by scholarship that examines how institutional trajectories shape the production and recognition of employable dispositions. Studies of schooling and higher education in South Africa demonstrate that attributes such as confidence, linguistic fluency, and interactional ease are cultivated through differentiated educational pathways, including historically advantaged schools and universities. Hunter (2019, 2020) analysis of white tone and educational differentiation shows how markers of professionalism are institutionalised through schooling and are subsequently read as indicators of competence in labour market settings. Crucially, Hunter illustrates that such dispositions are not reducible to race alone but emerge through the intersection of race, class, and institutional location. It also highlights how whiteness increasingly operates as a cultural and institutional formation that can be performed across racial groups, complicating binary distinctions between Black and white candidates while leaving underlying hierarchies intact. Relatability, in this sense, can be understood as an evaluative mechanism through which these institutionally produced dispositions are recognised and valorised in hiring contexts.

This line of analysis can be extended through engagement with broader South African scholarship on labour market stratification and employability. Webb's (2021) work on spatial stigma highlights how township‐based youth are not only economically marginalised but also symbolically positioned as less desirable workers, with place‐based identities shaping employer expectations and hiring decisions. Such spatialised perceptions intersect with cultural and linguistic markers, reinforcing the association between employability and proximity to historically advantaged urban and suburban spaces. Similarly, Jeske's (2020) ethnographic work demonstrates how employer narratives construct young job seekers as either motivated and deserving or lacking work ethic and discipline. These moralised distinctions often map onto classed and racialised assumptions, shaping how candidates are interpreted in hiring encounters. Together, this body of work underscores that employability is not simply a function of skills or qualifications, but is mediated through culturally and institutionally produced narratives of worth, readiness, and belonging. Building on these insights, we conceptualise relatability as the interactional mechanism through which these spatial, cultural, and moral distinctions are enacted in hiring processes. Relatability operates as a filter that translates historically produced inequalities in schooling, geography, and socialisation into judgements of fit, competence, and organisational safety.

These processes reproduce advantage across generations, even in the presence of formal equality and transformation frameworks, including employment equity and B‐BBEE legislation (Republic of South Africa 1998, 2003). Importantly, such inequalities are material and cultural in form, shaping dispositions, aspirations, and interactional styles that are later evaluated in institutional settings. In this context, relatability can be understood as an evaluative mechanism through which these structurally produced differences are recognised, misrecognised, and acted upon in hiring processes. Importantly, these dynamics unfold not only between racial groups but also within them. Post‐apartheid South Africa has witnessed the expansion of a Black middle class alongside persistent structural disadvantage, generating stratification within racial groups in access to cultural and linguistic capital. As a result, markers of employability increasingly reflect classed institutional trajectories, including schooling type, language use, and exposure to professional environments. Relatability therefore operates through the intersection of race and class, even as dominant standards of professionalism remain historically anchored in whiteness.

Against this backdrop, research on hiring discrimination illuminates how inequality is reproduced through ostensibly neutral procedures. Critical race scholarship shows how institutional norms sustain racial hierarchies despite formal equality commitments (Ali 2022; Delgado and Stefancic 2017). Under informational uncertainty, statistical discrimination models anticipate reliance on group‐based heuristics (Phelps 1972), while taste‐based accounts trace the persistence of prejudice (Becker 1957). In contemporary firms, overt bias is frequently recast as fit, merit and professionalism (Rivera 2012). Recent evidence suggests that while racial disparities in hiring outcomes may narrow higher up organisational ladders, they rarely disappear; in some cases, minoritised candidates are valued primarily for diversity optics rather than substantive inclusion (Heath and Di Stasio 2019; Weisshaar et al. 2024). For post‐apartheid South Africa, this shift is consequential: the rhetoric of meritocracy can convert historically accumulated advantage into the language of ‘readiness’ and ‘fit’, rendering exclusion harder to name and to regulate (Branson et al. 2024).

A complementary body of work in social psychology and sociology traces the influence of unconscious bias and homophily on evaluative judgements. Studies of implicit bias show evaluators favouring those with whom they share salient characteristics, often outside awareness (Greenwald and Krieger 2006). Network research on homophily demonstrates how shared educational, cultural and class backgrounds channel opportunities within relatively closed circles (McPherson et al. 2001). Field experiments reveal that otherwise identical CVs elicit different responses based solely on names or subtle signals, and that the effects of diversity training decay quickly, with discriminatory behaviours resurfacing within months (Madera and Hebl 2012; Pager et al. 2009). Research indicates that hiring managers continue to invoke culture fit to rationalise similarity preferences (Tholen 2024). In South Africa, where segregated schooling and neighbourhoods have long shaped social worlds, these seemingly benign preferences for the familiar intersect with racialised life trajectories, hardening advantage for some while quietly disqualifying others.

A further strand, on aesthetic labour and respectability politics, foregrounds the role of embodied dispositions in employability judgements. Employers increasingly seek workers who ‘look good and sound right’ (Warhurst and Nickson 2020). Read through Bourdieu's account of cultural capital, attributes such as accent, dress, posture and conversational ease are better understood as products of long socialisation, rather than as innate merit (Anýžová and Matějů 2018; Bourdieu 1984). Professional appearance standards, presented as neutral, often reproduce racialised, gendered and classed hierarchies (Dubois and Pansu 2004; Waters 2025). In practice, markers associated with white, middle‐class comportment are misrecognised as universal professionalism, rewarding those already socialised into these codes and requiring others to ‘adapt’, in ways that legitimate the codes themselves; an alignment with South African hiring narratives in which ‘client‐readiness’ and ‘polish’ are routinely valorised.

Emerging technologies introduce additional complexity. AI‐enabled screening tools can entrench historical inequities via biased training data and design choices; while carefully engineered systems may dampen some human prejudices, the scope for algorithmic discrimination remains substantial (Özbilgin et al. 2025). Jurisprudence is evolving in parallel—with rulings that discriminatory statements related to recruitment can constitute unlawful discrimination even outside formal hiring processes (European Court of Justice 2008). In a labour market whose historical data are racially skewed, automation risks hard‐coding yesterday's exclusions into tomorrow's filters, unless explicit fairness constraints, auditing and accountability mechanisms are built in, an especially pressing concern where graduate pipelines and internship platforms already reflect unequal access.

These distinct strands of scholarship leave significant gaps in explaining youth exclusion from employment. Macro‐level accounts clarify structural constraints; micro‐level studies illuminate interpersonal and organisational dynamics. What remains underspecified is the connective tissue, that is, the mundane practices and interpretive routines through which structure is enacted in ordinary hiring talk and everyday screening. Global evidence mapping underscores the imbalance, youth employment research is concentrated in high‐income countries despite Africa having the largest NEET population and the greatest need for intervention research (Apunyo et al. 2022). South Africa therefore requires inquiry that can surface the lived enactment of exclusion and situate it within specific socio‐historical conditions, an analytic task well served by combining hermeneutic phenomenology's interpretive sensitivity with grounded theory's systematic theory‐building.

## 3 Methods

We adopted a qualitative interpretivist approach grounded in hermeneutic phenomenology and supported by grounded theory analytical procedures (Annells 2006; Urcia 2021; Folgueiras Bertomeu and Sandín Esteban 2023). To enhance readability, we present only the core elements of research design and analysis, while detailed procedural steps are available from the authors upon request. The hermeneutic dimension of our approach treats understanding as emerging through interpretive dialogue between researcher and participants, recognising that meaning is contextually and historically situated (Laverty 2003). This orientation is well suited to examining how structural inequalities are expressed through everyday employer interactions and sense‐making processes, particularly where discrimination operates through coded language and implicit bias. We integrate hermeneutic phenomenology with grounded theory procedures to combine systematic coding with interpretive sensitivity in analysing participants' accounts (Laverty 2003; Charmaz 2014). While several analytical steps are illustrated through tables for transparency, these are intended as exemplars rather than exhaustive procedural documentation.

Existing sociological research has documented cultural matching, homophily, and aesthetic labour in hiring processes (McPherson et al. 2001; Rivera 2012; Warhurst and Nickson 2020). What remains less examined is how these evaluative tendencies operate together as a coherent mechanism through which employers manage uncertainty, risk, and organisational comfort in recruitment decisions (Roscigno et al. 2007; Tholen 2024). By focussing on relatability as an everyday evaluative practice, this article links interactional judgements to broader structures of racial inequality, showing how seemingly mundane hiring decisions contribute to the reproduction of labour market exclusion (Bonilla‐Silva 1997; Bourdieu 1984).

### 3.1 Sampling Strategy

We employed purposive sampling to recruit participants who could provide rich insights into racialised hiring practices. Such an approach enabled the identification and selection of information‐rich cases related to the phenomenon of interest, ensuring alignment between our sampling strategy and phenomenological research methodology when investigating racialised employment experiences (Birzer and Smith‐Mahdi 2006). Our sampling strategy prioritised conceptual depth over statistical representation, recognising that qualitative research does not require specific sample sizes determined a priori, as sample size should only be considered in relation to research purpose, chosen methodology, and sample composition (Vasileiou et al. 2018). Through this methodology, we captured how employment decision‐making processes unfold across different organisational contexts and stakeholder positions. The study is therefore analytically focused on corporate hiring processes associated with graduate and early‐career recruitment, rather than the labour market as a whole.

A total of 13 participants were recruited, representing three distinct stakeholder groups positioned to illuminate different aspects of the youth employment landscape (Table 1). The core group comprised 10 senior human resources professionals and hiring managers from South African corporates, each with extensive experience in youth recruitment spanning both matriculant and graduate levels. These employer participants collectively possessed extensive experience spanning both matriculant and graduate recruitment, with each having a minimum of 15 years in human resource management or recruitment roles. Their industry experience was diverse, encompassing financial services, telecommunications, consumer goods, logistics, professional services, and engineering consulting sectors. All participants held senior positions with substantial influence over recruitment decisions, ranging from HR specialists to C‐suite executives, with company sizes varying from 350 to over 50,000 employees, representing both national and multinational operations. Their collective experience spanned diverse industries including banking, insurance, telecommunications, consumer goods, logistics, professional services, and engineering consulting. The demographic composition of our sample is aligned with South Africa's complex racial landscape, with participants including Black African, Indian, and White professionals. The gender distribution in our sample, which is weighted toward women, reflects the composition of senior human resource and recruitment roles in the organisational contexts accessed, where women are often overrepresented. At the same time, we do not treat this distribution as representative of the wider labour market, but as a feature of the specific organisational field under study. The sample is not intended to be statistically representative; rather, it is purposively constructed to capture information‐rich perspectives from actors directly involved in corporate hiring processes. This diversity proved analytically significant, as it enabled us to examine how different racial positionalities within corporate hierarchies might influence hiring perspectives and decision‐making processes. Table 1 provides an overview of participants' demographic information. Consistent with qualitative research design, our aim is analytical rather than statistical generalisation, prioritising depth of insight into hiring practices over demographic representativeness.

**TABLE 1:** Participants' demographics.

Pseudonym	Participant type	Gender	Age	Race	Position/Role	Industry sector	Years of experience	Company size (employees)	Geographic scope
Jill	Corporate employer	Female	46–50	White	Senior talent sourcing specialist	Financial services	18+	10,000–50,000	National
Preggie	Corporate employer	Male	51–55	Black African	C‐suite executive	Multi‐sector	25+	5000–15,000	Multi‐national
Shamala	Youth work‐readiness intermediary	Female	46–50	Indian	C‐suite executive	Youth development	20+	500–2000	National
Nomonde	Corporate employer	Female	41–45	Black African	Divisional head of HR	Consumer goods	15+	10,000–50,000	Multi‐national
Tshego	Corporate employer	Female	41–45	Black African	Associate director HR	Consulting	15+	1000–5000	Multi‐national
Kumaran	Employer Federation representative	Male	46–50	Indian	Senior executive	Multi‐industry federation	20+	N/A (Federation)	National
Fundiswa	Corporate employer	Female	41–45	Black African	Head of HR	Telecommunications	15+	2000–10,000	National
Puleng	Corporate employer	Female	41–45	Black African	HR director	Specialised financial services	15+	1000–5000	National
Valerie	Corporate employer	Female	41–45	Black African	Head of HR	Telecommunications	18+	5000–15,000	National
Reshma	Corporate employer	Female	46–50	Indian	Strategic HR manager	Financial services	18+	5000–15,000	National
Lebo	Corporate employer	Female	46–50	Black African	Senior group leadership & HR development specialist	Logistics	x18+	10,000–50,000	Multi‐national
Lorna	Corporate employer	Female	36–40	Black African	HR director	Consumer goods	15+	5000–15,000	Multi‐national
Ravesh	Youth work‐readiness intermediary	Male	56–60	Indian	HR director	Engineering consulting	20+	350–1000	Multi‐national

The analytical focus on employers and labour market intermediaries is deliberate. By centring decision‐makers’ accounts, the study examines how labour market exclusion is actively produced through hiring judgements, rather than inferred solely from worker outcomes. This demand‐side perspective allows us to trace how inequality is enacted through organisational routines and everyday evaluative practices.

### 3.2 Data Collection

The study received ethical approval from the lead author's University's Commerce Faculty Ethics Committee, with particular attention paid to the sensitive nature of research exploring racial attitudes and employment discrimination. All participants provided written informed consent after receiving detailed information about the study's purposes, methods, and potential implications. We emphasised voluntary participation and the right to withdraw at any stage, recognising that discussions of racial preferences in hiring might generate discomfort among participants.

It was not our intention to adopt conventional interview formats, but to create encounters that functioned as spaces for exploring how participants construct their hiring preferences through dialogue. Following critique of traditional interview approaches, we considered our encounters with employers as opportunities to understand how they constitute their decision‐making logics in the very process of articulating them.

This study employed a semi‐structured interview protocol consisting of predetermined questions with flexibility for follow‐up probes, specifically crafted to navigate the sensitive terrain of racial attitudes and unconscious bias in employment practices. The protocol structure included four distinct sections: opening remarks (study introduction and participant briefing), warm‐up questions (role/position and youth employment experience), core thematic enquiries (recruitment attributes, racial disparities, decision‐making processes, and cultural factors), and concluding remarks with demographic information, utilising both direct and oblique questioning techniques to elicit explicit reasoning and implicit assumptions from employer participants.

Semi‐structured interviews were used to balance consistency with flexibility in probing participants' responses (DeJonckheere and Vaughn 2019). Questions explored how participants made sense of racial disparities in youth employment outcomes, their hiring preferences and practices, and their interpretations of organisational culture and candidate suitability. We combined direct questions on recruitment processes with more oblique prompts to elicit both explicit reasoning and tacit assumptions, recognising that participants may not always be fully aware of, or willing to articulate, potentially discriminatory preferences. Each interview lasted approximately 1 hour and was conducted either face‐to‐face or via Microsoft Teams, depending on participant preference. All interviews were audio‐recorded with consent and transcribed verbatim to preserve linguistic nuance for analysis.

### 3.3 Analysis

We were guided by an hermeneutic approach to our analysis, seeking first to understand participants' experiences on their own terms. This initial reading then informed an iterative dialogue between the data generated in our encounters and relevant theoretical frameworks. In contrast to a purely inductive logic that builds theory from a blank slate, our analysis began with a puzzling empirical reality: the persistent employment disadvantage faced by Black African youth. Our analytical aim was thus to generate the most plausible theoretical explanation for this phenomenon. This process entailed a constant, reflexive movement between our empirical data and emergent theoretical constructs, allowing one to inform and shape the other. This hermeneutic process, a cornerstone of many contemporary grounded theory techniques, allowed us to construct a coherent framework that acknowledges the researcher's role in theory construction, while ensuring both methodological rigour and a deep sensitivity to the meanings constituted in our participants' lived experiences.

Analysis proceeded through the innovative and systematic strategy of simultaneous data collection and analysis characteristic of grounded theory approaches (Chun Tie et al. 2019). Our analytical process was designed to maintain transparency and traceability while navigating the rich qualitative data from our encounters. The primary analytical step involved open coding, where we meticulously worked through the transcribed texts. Open coding generated initial categories grounded in participants' language, which were refined through constant comparison (Taherdoost 2022). Initial codes were iteratively grouped into higher‐order categories. An example of this initial categorisation, showing how individual codes are clustered under broader themes, is visually represented in our study's figures. As an illustrative example of this analytical progression, Table 2 demonstrates how participants' exact language was systematically abstracted into theoretical constructs while maintaining clear empirical grounding, showing the complete mapping for the core category ‘confidence’ where 50 initial codes were grouped into five substantive categories before being integrated into a single theoretical construct that captures the racialised nature of confidence expectations in hiring processes.

**TABLE 2:** Illustrative example of three‐stage analytical progression from initial codes to core category.

Initial codes	Substantive categories	Core category
‘Cocky confidence’, ‘assurance in presentation’, ‘white applicant confidence’, ‘certain humility on black applicants’, ‘quiet out of respect’, ‘apologetic manner of showing up’, ‘don't feel they can be themselves’, ‘respect thing or culture thing’, ‘not groomed to have a voice’, ‘don't care what next person thinks’	Cultural expression of confidence	Confidence
‘Comfortable in adult spaces’, ‘intimidated for that moment’, ‘feel like this is not their real home’, ‘can't afford to be shy’, ‘battle with basic things’, ‘feel free, feel comfortable’, ‘elevated themselves’, ‘level of showing up’, ‘apologetic kind of manner’, ‘cultural competence required’	Social positioning and comfort	
‘Ability to express and present’, ‘hold their own in conversation’, ‘talk as if they know what they're talking about’, ‘walk in with this set thing’, ‘exuding confidence in interview’, ‘cannot articulate themselves’, ‘struggle to express themselves’, ‘completely went pear‐shaped’, ‘nerves affecting performance’, ‘comfortable conversation flow’	Interview performance dynamics	
‘Model C confidence levels’, ‘multi‐racial school presentation’, ‘private school assurance’, ‘affluent area exposure’, ‘lower LSM hesitation’, ‘township background uncertainty’, ‘disadvantaged background impact’, ‘Bantu education limitations’, ‘social standing influence’, ‘economic background effect’	Class‐based confidence markers	
‘Something about him missing’, ‘what is something exactly’, ‘confidence that we're looking for’, ‘professional brand selling’, ‘client‐facing confidence’, ‘polished presentation expected’, ‘caliber based on confidence’, ‘potential versus interview skills’, ‘mature interviewer needed’, ‘cultural competence to recognize’	Employer perception of confidence	

To move from descriptive categories to a coherent theoretical framework, we used analytical tools to deepen interpretive engagement. Chief among these was the conditional relationship guide, which maps the conditions, contexts, interactions, and consequences associated with each category to reveal how exclusion mechanisms interact (Charmaz 2014). Table 3 illustrates how categories such as self‐presentation and confidence operate interdependently, showing when cultural misreadings of deference as incompetence occur and how they produce systematic disadvantage for Black African candidates.

**TABLE 3:** Conditional relationship guide: systematic interrogation of connections between categories.

Categories	Conditions (when/Why)	Contexts (where)	Interactions (how)	Consequences (what results)
Self‐presentation → Confidence	When candidates lack ‘appropriate’ aesthetic markers or communication styles expected in corporate settings	Interview situations, client‐facing roles, professional service environments	Employers interpret cultural communication patterns as lack of confidence, misread deference as weakness	Systematic undervaluation of black African candidates, cultural gatekeeping reinforced
Bias → Choice	When time pressure combines with unconscious preferences for familiar cultural markers	Competitive hiring markets, resource‐constrained recruitment processes	Employers resort to ‘gut feel’ decisions, rely on referral networks, minimise perceived risks	Homogeneous hiring patterns, network reproduction, systematic exclusion of outsiders
Affinity → Bias	When employers unconsciously gravitate toward candidates with shared cultural experiences	Organisational cultures dominated by specific racial/class groups	Recognition processes favour familiar social codes, comfort levels guide selection decisions	Social closure mechanisms activated, diversity initiatives undermined
Confidence → Self‐presentation	When culturally‐coded expectations about assertiveness conflict with candidates' socialisation patterns	Corporate environments valuing individual assertiveness over collective respect	Employers misinterpret cultural humility as professional inadequacy, expect white middle‐class presentation norms	Competent candidates excluded based on cultural presentation rather than ability
Choice → Affinity	When employers seek ‘safe’ hiring decisions under market pressures	High‐stakes recruitment situations, leadership pipeline positions	Decision‐makers activate homophily preferences, prioritise cultural fit over competence	Elite position reproduction, institutional whiteness maintained

The final stage of analysis involved a reflective coding matrix. Drawing on hermeneutic phenomenology and grounded theory (Laverty 2003; Annells 2006; Urcia 2021), categories were organised by their properties, processes, and consequences to examine how employer sense‐making constructs barriers to employment. This matrix supported selective coding (Charmaz 2014) and enabled the identification of a unifying relational filter linking aesthetic impressions, communication norms, perceived readiness, similarity preferences, and implicit bias. Table 4 maps these categories across analytical dimensions, demonstrating how they collectively reproduce racialised exclusion.

**TABLE 4:** Reflective coding matrix: properties, processes, and consequences.

Core categories	Properties	Processes	Conditions	Contexts	Interactions	Consequences
Self‐presentation	Aesthetic conformity, communication style, professional demeanour, cultural capital display	Visual assessment, linguistic evaluation, behavioural coding, status signalling	White organisational norms, class‐based standards, educational background	Interview settings, corporate environments, client‐facing roles	Employer judgement, candidate performance, cultural (mis)match	Inclusion/exclusion decisions, competence assumptions, cultural gatekeeping
Confidence	Cultural coding, social positioning, comfort levels, Assertiveness display	Performance evaluation, cultural interpretation, behavioural assessment	Racialised socialisation, educational privilege, class background	Interview dynamics, power relations, cultural expectations	Misrecognition of deference, cultural misreading, bias activation	Systematic disadvantage, talent misidentification, cultural discrimination
Bias	Conscious awareness, institutional embedding, justification mechanisms	Unconscious activation, rationalisation, denial processes	Historical legacies, structural inequalities, organisational culture	Recruitment processes, decision‐making moments, evaluation criteria	Implicit judgements, stereotype activation, preference formation	Discriminatory outcomes, pattern reproduction, system maintenance
Choice	Risk minimisation, efficiency priorities, intuitive selection, convenience focus	Gut‐feel decisions, referral reliance, shortcut usage, quick judgements	Market pressures, time constraints, resource limitations	Competitive environments, scarcity contexts, business pressures	Employer preferences, network activation, familiarity seeking	Homogeneous hiring, network reproduction, exclusionary patterns
Affinity	Cultural proximity, shared experiences, comfort zones, familiarity preferences	Homophily activation, similarity recognition, comfort seeking, bonding assessment	Social backgrounds, cultural socialisation, educational experiences	Social interactions, workplace dynamics, team integration	Recognition patterns, comfort levels, chemistry evaluation	Social closure, cultural reproduction, diversity limitation

Table 5 complements the reflective coding matrix by providing a focused examination of each core category's constituent properties and dimensional ranges, offering a detailed conceptual foundation for understanding how these interlocking processes operate through multiple, mutually reinforcing pathways. This structured and reflexive approach provided a clear and defensible audit trail, documenting the interpretive path from raw participant accounts to the development of our final theoretical model.

**TABLE 5:** Thematic analysis framework: core categories and properties.

Core category	Properties	Dimensional range
Self‐presentation	Aesthetic conformity	Polished ←→ Unpolished
	Communication style	Articulate ←→ Inarticulate
	Professional demeanour	Confident ←→ Uncertain
Confidence	Cultural coding	Assured ←→ Apologetic
	Social positioning	Assertive ←→ Deferential
	Comfort level	Entitled ←→ Grateful
Bias	Conscious awareness	Explicit ←→ Unconscious
	Institutional embedding	Individual ←→ Systemic
	Justification logic	Acknowledged ←→ Denied
Choice	Decision rationale	Scientific ←→ Intuitive
	Risk assessment	Developmental ←→ Ready‐made
	Effort minimisation	Inclusive ←→ Selective
Affinity	Cultural proximity	Familiar ←→ Foreign
	Shared experience	Similar ←→ Different
	Comfort zones	Relatable ←→ Alienating

### 3.4 Methodological Rigour and Reflexivity

We implemented multiple strategies to ensure methodological rigour following established frameworks for qualitative research quality. Data source triangulation across three stakeholder groups provided different perspectives on the central phenomenon, while detailed documentation of analytical procedures created an audit trail for evaluating our interpretive processes.

Researcher reflexivity proved particularly critical given the sensitive nature of racial discrimination research. The lead researcher, a Black South African with extensive human resources experience, brought valuable insider knowledge of corporate hiring dynamics while requiring careful attention to potential bias and over‐identification with participants. The research team's diverse composition provided multiple analytical perspectives and enhanced our capacity to interrogate findings across different experiential and theoretical lenses.

Regular analytical memos and supervisory discussions documented how personal experiences and theoretical commitments might influence data interpretation. The research team engaged in ongoing reflexive dialogue throughout the analytical process, ensuring that individual positionalities enriched rather than constrained the interpretive process. This reflexivity was central to our understanding that knowledge production in this domain is inevitably shaped by researchers' own social locations and experiences of racialised labour markets.

The study does not include organisational recruitment datasets, such as applicant pools, shortlists, or hiring outcomes. While such data would enable further triangulation, our qualitative approach is designed to capture the interpretive logics through which hiring decisions are made. Future research could usefully integrate organisational data to examine how these evaluative processes translate into measurable outcomes.

### 3.5 Findings

Guided by the hermeneutic‐grounded strategy, our findings offer an analytic narrative of employer reasoning. The focus rests on the patterned routines through which everyday hiring talk hardens into systematic exclusion. Rather than being determined solely by formal criteria such as qualifications or experience, participants' narratives revealed how perceptions of interpersonal ease, organisational fit, and reputational safety shaped evaluations long before competency metrics were formally applied. These early judgements acted as powerful filters, hiring choices were often guided by implicit assessments of candidate comfort, perceived readiness, and social familiarity, factors tightly intertwined with classed and racialised assumptions that disadvantage Black African youth in particular. These processes are not reducible to race alone; rather, they operate through classed and spatial distinctions within racial groups, reflecting differences in schooling, geography, and institutional trajectories that shape access to dominant norms of professionalism.

Participants articulated five interlocking processes that constitute this exclusionary calculus: (1) self‐presentation, (2) confidence, (3) bias, (4) choice, and (5) affinity. Each process may appear neutral in isolation, yet together they operate as a relational filter that rewards proximity to whiteness (Al Ariss et al. 2014) and penalises cultural or socio‐economic divergence. The sections that follow examine each process in turn, showing how they operate in practice and how they collectively reinforce racialised closure beneath a meritocratic veneer.

### 3.6 Self‐Presentation as a Racialised Construct

First impressions in South African recruitment are rarely neutral; instead, they are saturated with aesthetic and classed expectations that shape whether a candidate is seen as client‐ready. Drawing on aesthetic‐labour scholarship (Warhurst and Nickson 2020) and racialised respectability politics (Johnson 2013), we conceptualise self‐presentation as a pivotal gatekeeping ritual. Clothing, posture and embodied comportment serve as shorthand signals of professional legitimacy, enabling employers to sift applicants before technical competencies are even considered. Aesthetic judgements are benign and intertwined with colonial legacies and racial hierarchies, functioning as seemingly neutral proxies for broader social inequalities (Adisa et al. 2024; Umeh et al. 2024). Our analysis aligns with this, revealing how South African hiring panels interpret embodied cues, such as grooming, stance, and tone of voice, through a middle‐class, often white‐coded aesthetic register. Candidates who deviate from this standard face silent disqualification under the guise of professionalism. Our participant, Nomonde, is a Black African divisional head of HR in a multinational consumer goods conglomerate. Raised through the graduate‐trainee pipeline, she now manages her team, with a remit that includes grooming entry‐level talent for brand‐sensitive client roles. This experience shapes her vigilance over interview décor and posture. This racialised reading of decorum renders Black African youth, particularly those from resource‐constrained settings, inherently less relatable in aesthetic terms:

‘How do you present yourself in the initial interview?’ And that's a big question. And the reality is, I don't know if I want to call them millennials, when sometimes they come into an interview, it's not about wearing a suit or not wearing a suit. It's also about your posture, it's about your confidence, it's about knowing that certain attires are not to be worn, obviously in an interview. It doesn’t have to be a suit. You can wear just plain black and white. You can see somebody who's prepared and who's been taught versus somebody who just comes in a crop top and a very revealing top. And now, let's say you, unfortunately you're interviewing with actuaries, who are sticklers for these things. And the first thing that comes to their minds, ‘Oh my God. This person is going to go see our clients’.

(Nomonde, 41–45, Female, Black African, Divisional Head of HR, Consumer Goods, 15+ years)

Nomonde's commentary reveals how organisational guardians translate seemingly neutral dress codes into risk assessments about client exposure in ways that align with broader patterns of aesthetic discrimination documented in hiring research (Ruggs et al. 2013). In practice, the yardstick is calibrated against white‐middle‐class norms of modesty and composure; those schooled outside that habitus arrive already ‘out of place’ and are read as jeopardising the firm's commercial image. Reshma, an Indian strategic HR manager at a national financial services group, oversees flagship graduate intakes. Her upward mobility from a lower‐LSM background informs a paradoxical stance: she is empathetic to resource constraints yet still tasked with defending corporate aesthetics. What appears to be an evaluation of professionalism is, a classed and racialised proxy for corporate safety:

Unfortunately, those coming from the lower LSM, so previously disadvantaged backgrounds, do have social and economic factors that influence their personal brand. Dress attire is one of them. From my experience on the graduate side, they often have hand‐me‐down corporate clothes that they wear—sometimes too big, too loose, too tight. Unfortunately, that is all they have available.

(Reshma, 46–50, Female, Indian, Strategic HR Manager, Financial Services, 18+ years)

Reshma's reflection exposes a structural bind: candidates most distant from the historically white corporate style are judged against it, while the organisation disclaims responsibility for unequal sartorial resources. Thus, self‐presentation operates less as an individual skill than as a screening device that converts inherited material disadvantage into a narrative of personal unreadiness.

These accounts demonstrate that corporate dress norms serve as a covert sorting device: polished, well‐tailored attire is interpreted as evidence of professionalism and client safety, whereas hand‐me‐down suits and unfamiliar posture translate material disadvantage into a narrative of personal unreadiness, allowing racial‐class hierarchies to be reasserted long before skills are even discussed.

### 3.7 Confidence as a Racialised Construct

Recruiters framed an assured conversational style, for instance steady eye contact, quick humour, fluid small talk, as a proxy for initiative and leadership potential. Building on work on confidence culture (Orgad and Gill 2021) and racialised embodiment (Ahmed 2021), we read confidence not as an innate trait but as a learnt display shaped by schooling, social networks and comfort in elite spaces. Through such displays, seemingly minor cues, including tone, cadence, ease of movement, signal membership of a world where self‐promotion is customary, leaving quieter forms of competence unheard. One of our participants, Puleng, a Black African HR director in a specialist finance house employing about three thousand staff, described the instant authority White graduates appear to wield when they enter an interview room:

I have to tell you, there’s this cocky confidence that White applicants have and I can’t explain it. There’s assurance, a confidence—they walk in with this sense that ‘I’ve got the job’—and a certain humility on Black applicants that sometimes actually doesn’t work for them. If you are not culturally competent, it’s easy to overlook the person who is more humble and quiet out of respect, because you enjoyed the conversation with the other person so much.

(Puleng, 41–45, Female, Black African, HR Director, Specialised Financial Services, 15+ years)

Puleng's description points to how ease with self‐assertion serves as a cultural marker of belonging. The fast, assured greeting performs readiness; the subdued courtesy of many Black interviewees registers as uncertainty. In the appraisal moment, what matters is less what is said than the confident rhythm with which it is delivered, an interview dynamic that converts familiarity with middle‐class social codes into an aura of competence. Here, racialised dispositions become misread as personal deficits, amplifying exclusion under the guise of presence. A parallel hierarchy surfaces in Fundiswa's account. Now heading HR in a national telecommunications firm with roughly 7000 employees, she has guided numerous township‐raised graduates through assessment centres, but still sees conversational flair tipping the scales. Black African graduates, even when academically qualified, thus face a relational mismatch with the dominant performance script:

There’s a certain level of confidence you get from White boys and girls—their ability simply to hold a conversation. Even if it’s mediocre, they talk as if they know what they’re talking about, whereas they know nothing. Our Black youngsters haven’t been groomed to have a voice; they show up almost apologetically.

(Fundiswa, 41–45, Female, Black African, Head of HR, Telecommunications, 15+ years)

Fundiswa identifies the same pattern: fluency and relaxed banter, fostered in well‐resourced schools and dinner‐table debates, travel into the interview as evidence of intellect. Where class affords practice in public speaking, confidence becomes a visible commodity; where economic marginalisation curtails such rehearsal, hesitancy is read as lack of promise. Employer impressions are thus shaped less by technical substance than by how comfortably an applicant occupies the room, reinforcing a hiring calculus in which polished self‐projection confirms prior expectations of merit.

### 3.8 Racial Bias

Hiring decisions were often narrated as objective, yet closer inspection revealed a preference for candidates who mirrored the recruiter's own educational, linguistic and cultural background, a pattern consistent with theories of homophily (McPherson et al. 2001) and unconscious bias (Greenwald and Krieger 2006). This tendency is not confined to anecdote: South African recruiters exhibit systematic preferences for candidates who share similar backgrounds, perpetuating post‐apartheid inequalities even when controlling for qualifications (Branson et al. 2024). Such leanings operate quietly, legitimised by talk of fit or proven credentials, and convert resemblance into a proxy for reliability. Lorna, a Black African HR director in a multinational consumer‐goods firm, who oversees mass graduate intakes for production and supply‐chain roles, described how, despite formal diversity commitments, shortlisting often gravitates toward applicants whose speech patterns, demeanour, and alma mater match those already in the organisation. Balancing equity targets with the pace of high‐volume staffing, she reflects on how implicit filters seep into routine shortlisting:

Why are Black kids the most unemployed? Because human beings cannot remove their biases from recruitment. Unfortunately, those biases are unconscious. Sometimes we’re not even aware of the biases we apply while recruiting.

(Lorna, 36–40, Female, Black African, HR Director, Consumer Goods, 15+ years)

At the same time, other participants explicitly recognised these tendencies, describing them not as errors but as practical necessities in high‐pressure hiring environments. Lorna's observation points to a tacit ranking system in which accent, demeanour and alma mater carry hidden weight. The acknowledgment that ‘we're not even aware’ gestures to selection panels silently discarding candidates whose manner deviates from an internal standard, an example of bias masked as neutral calibration. These imperceptible preferences silently recalibrate merit through a lens that privileges whiteness and middle‐class familiarity. Reshma serves as a strategic HR manager in a national financial‐services group, steering selection for specialist risk and actuarial posts. With nearly 2 decades in talent acquisition, she foregrounds the magnetism of similarity when vacancies arise:

One thing we find is that individuals like hiring people who are like them—same schooling, same background, same experience. Managers tend to replace the person who left rather than fill the job, so if the previous person had an MBA or spoke Afrikaans, they look for that again even when the role doesn’t require it.

(Reshma, 46–50, Female, Indian, Strategic HR Manager, Financial Services, 18+ years)

Reshma's description highlights how the quest for a seamless handover turns prior incumbents into templates, prompting selectors to favour matching degrees, languages and social cues, even when those attributes outstrip job requirements. Credentials become shorthand for predictability, enabling managers to feel safe while perpetuating a cycle in which historically advantaged profiles remain the default choice and Black African youth are evaluated against benchmarks never designed for them. The reproduction of such standards embeds exclusionary pathways into recruitment processes, systematically disadvantaging those whose social trajectories diverge from the dominant script.

### 3.9 Choice as a Racialised Concept

Recruiters routinely framed selection as a matter of operational efficiency: the closer a candidate came to immediate productivity, the more attractive they became. Echoing theories of risk‐averse human‐capital deployment (Coff and Kryscynski 2011), choice hinged on who would demand the fewest supervisory resources, generate the least litigation threat and integrate fastest into existing workflows, the core of the ‘plug‐and‐play’ mentality that privileges perceived readiness over developmental potential. Preggie, a Black African C‐suite executive with over 25 years of experience in multi‐sector leadership, supervises large project portfolios subject to tight cost controls. His calculus for graduate intake is blunt:

If I’m going to hire five people, I want the one who is going to be the least hassle for me—someone who already knows what to do. I don’t have time or supervisors running around; I can’t carry all this cost. So I choose the person who is more ‘ready’, with a CV and good references, rather than hire someone completely misplaced who then takes me to the CCMA.

(Preggie, 51–55, Male, Black African, C‐Suite Executive, Multi‐sector, 25+ years)

Preggie's comments reveal how perceived self‐sufficiency and reputational safety eclipse broader inclusion aims. The spectre of CCMA disputes and the desire to avoid ‘running around’ recast developmental hires as liabilities; financial and legal risk is offloaded onto those lacking prior exposure, reinforcing a preference for candidates already socialised into corporate routines. This cost‐conscious logic operates as a racialised filtering tool, quietly disfavouring Black African graduates who lack familiarity with the performative codes and organisational behaviours prized in dominant corporate settings. The same calculus shapes Jill's approach. As a white senior talent‐sourcing specialist in a national financial‐services group, she curates flagship graduate programmes designed to feed high‐yield business units:

Companies are going to take the route of ‘let’s take someone who’s ready‐baked.’ It’s easy plug‐and‐play, so that’s absolutely happening on flagship graduate programmes—though perhaps it’s controversial to say you can’t always force companies to take the longer route.

(Jill, 46–50, Female, White, Senior Talent Sourcing Specialist, Financial Services, 18+ years)

Such accounts are particularly striking given that many of these organisations operate under formal transformation mandates, highlighting the persistence of informal evaluative criteria alongside regulatory compliance. Jill, a senior talent sourcing specialist in a national financial services group, oversees the company's flagship graduate programmes. For her, speed and seamless integration are decisive virtues. Within this logic, ‘ready‐baked’ status, typically signalled by prestigious internships, fluent command of corporate vernacular, and polished references, becomes shorthand for low onboarding friction. Candidates perceived as able to slot effortlessly into existing workflows are prioritised, while those requiring additional guidance, trial‐and‐error learning, or cultural acclimatisation are quietly filtered out. This operational preference reflects patterns of risk deflection long documented in South Africa's outsourced labour market, where insecure workers are rendered expendable to shield employers from reputational and legal exposure (Runciman and Hlungwani 2022). In practice, selection narrows to a managerial comfort zone in which class‐inflected indicators of preparedness are privileged over demonstrable growth potential. For Black African youth who have not had the opportunity to accumulate such signals, exclusion becomes both routine and rationalised under the language of operational pragmatism.

### 3.10 Affinity as a Racialised Construct

Recruitment panels frequently translated comfort and shared reference points into a verdict of fit, echoing homophily research that shows how social proximity can masquerade as merit (Rivera 2012). Selectors emphasised familiarity with cultural codes, including schools attended, conversational idioms, and leisure tastes, as evidence that a candidate would mesh seamlessly with team dynamics and client expectations. Tshego is a Black African associate HR director in a global consulting partnership. Drawing on this trajectory, she notes that her initial sense of a candidate's suitability often rests on whether their world feels recognisably close to her own, shaping decisions in subtle but consistent ways:

The hiring manager’s race doesn’t necessarily determine whom they hire. What influences the decision is social standing. I can relate to a candidate from a model‐C school far more than to someone from a very disadvantaged background. People are more inclined to go with the candidate whose world feels closer to their own—it’s about whether our worlds relate to each other.

(Tshego, 41–45, Female, Black African, Associate Director HR, Consulting, 15+ years)

This suggests that relatability is not anchored in shared racial identity, but in shared institutional and class trajectories that structure recognition within hiring encounters. This illustrates how relatability is mediated by classed life‐worlds within racial groups, where shared middle‐class trajectories, rather than race alone, structure recognition and comfort. Tshego's reflection reveals how affinity operates through tacit cues of lifestyle consonance, such as school slang, accent, and even weekend hobbies, rather than through overt racial matching (Hunter and Hachimi 2012). These cues reassure decision‐makers that onboarding will require minimal cultural translation, thereby converting shared middle‐class habitus into an informal hiring criterion. In this sense, relatability is not race‐neutral; it is coded through the embodied familiarity of middle‐class whiteness, even when enacted by Black selectors.

A similar pattern emerges in Valerie's account, where affinity is articulated in more direct terms through everyday preferences and interpersonal comfort. Valerie who heads talent pipelines at a national telecommunications provider with approximately six thousand employees, grew up in a suburban Black middle‐class family and now supervises graduate rotations that feed into customer‐facing roles. She distils the mechanism more bluntly:

I think it’s a human flaw: we like to work with people we like, people we can relate to, people who are somehow like us.

(Valerie, 41–45, Female, Black African, Head of HR, Telecommunications, 18+ years)

By casting preference for similarity as a universal instinct, Valerie normalises the practice of choosing candidates whose conversational rhythms and cultural references mirror the panel's own, reinforcing what recent research describes as the subtle operation of homophily as a natural selection mechanism that reproduces social inequalities in African employment contexts (April et al. 2013). That sense of instant resonance reassures managers that onboarding will involve little cultural translation, while applicants whose life‐worlds diverge from the dominant script are quietly labelled potential friction points. By framing exclusion as the benign by‐product of comfort, the racialised construction of relatability remains hidden in plain sight. Here, similarity becomes institutional currency, redeemable only by those already fluent in the codes of dominant social groups.

## 4 Discussion and Conclusion: Relatability as a Racialised Mechanism of Closure

In the South African context, the legacy of apartheid continues to shape labour market outcomes through enduring structural inequalities as well as embodied dispositions and interactional norms (Durrheim et al. 2011; Seekings and Nattrass 2005). Differential schooling systems, linguistic hierarchies, and racially patterned pathways into professional spaces have generated uneven access to confidence, ease, and cultural familiarity (Bourdieu 1984; Fataar 2012; Soudien 2012). Hiring judgements centred on relatability function as a mechanism through which these historically produced inequalities are carried into contemporary organisational decision‐making (Bonilla‐Silva 1997; Roscigno et al. 2007). In this sense, relatability does not emerge in a vacuum; it is anchored in a broader inequality of opportunity regime that shapes who acquires the cultural, linguistic, and interactional resources that are later interpreted as merit. South African scholarship shows that employability is shaped through educational differentiation (Hunter 2019, 2020), spatial stigma (Webb 2021), and moralised narratives of youth work‐readiness (Jeske 2020), all of which structure how candidates are perceived and evaluated in hiring contexts. The analysis shows that relatability operates as a racialised mechanism of social closure within South African hiring practices. Across the interconnected processes of self‐presentation, confidence, bias, choice, and affinity, employers consistently privilege proximity to white, middle‐class norms, with classed dispositions such as schooling, accent, and institutional familiarity mediating how racialised expectations are enacted in practice (Moodley et al. 2025). Although articulated through ostensibly neutral and meritocratic criteria, these judgements draw on historically sedimented hierarchies and reproduce exclusion even in organisations that formally endorse equality and diversity.

At the micro level, employer assessments are shaped by culturally coded expectations of professionalism, including assumptions about how competence should appear, sound, and be felt in interaction. Self‐presentation and confidence emerge as early screening devices, frequently preceding substantive evaluation of skills or experience. From this perspective, Bourdieu's (1984) concept of cultural capital offers critical insight. The interactional and aesthetic codes valorised in recruitment reflect embodied dispositions acquired through unequal educational trajectories, family environments, and early professional exposure. When immediate conformity to these codes is demanded, structural inequalities in socialisation are recast as individual deficiencies, framed as unreadiness or lack of fit (Flemmen 2013).

These evaluative standards are embedded within a racialised social system in which material, cultural, and symbolic resources remain unevenly distributed along racial lines (Bonilla‐Silva 1997). Within the South African corporate sphere, whiteness continues to operate as a normative cultural and institutional reference point, reproduced through educational and organisational pathways rather than confined to white individuals alone. Attributes such as ease and self‐assurance, commonly associated with white graduates, reflect prolonged exposure to environments that reward self‐promotion and familiarity with dominant institutional norms. Interpreting these attributes as indicators of merit reinforces patterns of preferential hiring.

While whiteness remains a normative reference point, its operation is mediated through classed institutional trajectories. In practice, this means that middle‐class cultural dispositions, rather than race alone, often serve as the immediate basis for evaluation, even as access to these dispositions remains racially structured. However, because access to these dispositions remains racially patterned in South Africa, class does not displace race but rather operates as one of the key mechanisms through which racialised inequality is reproduced. Relatability therefore reflects the co‐constitution of race and class, rather than a simple binary opposition between Black and white candidates.

Bias, choice, and affinity further consolidate these dynamics. Evaluative bias, often rationalised through notions of fit or readiness, becomes routinised within organisational processes. Preferences for candidates requiring minimal supervision or rapid integration privilege those already acculturated to corporate norms, who are disproportionately drawn from white and middle‐class networks. Affinity converts shared cultural references into perceptions of competence, consistent with sociological accounts of homophily (McPherson et al. 2001). Together, these processes enact social closure by regulating access to employment through culturally specific criteria (Roscigno et al. 2007).

Relatability proves especially consequential because it frames structural exclusion as natural preference. Employers frequently understand their decisions as meritocratic, even as evaluative criteria remain saturated with routinised racial and class assumptions, described by participants as forms of unconscious and, at times, reflexively acknowledged judgement. This helps to account for the limited impact of equity legislation on Black African youth unemployment, including among credentialed graduates (Republic of South Africa 1998, 2003; National Treasury 2023). In South Africa, policy frameworks such as Broad‐Based Black Economic Empowerment (B‐BBEE), employment equity legislation, and youth employment incentives have sought to expand access to labour market opportunities and promote demographic transformation. However, these interventions primarily target structural inclusion and representation, often leaving the cultural–affective evaluative processes that shape hiring decisions largely unaddressed. While such policies can increase the presence of Black candidates within recruitment pipelines, our findings suggest that they do not fully disrupt the informal criteria through which candidates are judged in practice. Relatability operates as a filtering mechanism at the point of evaluation, where cultural familiarity, ease of interaction, and perceived organisational fit continue to shape outcomes in ways that may undermine policy intentions. Therefore, structural reforms may expand access without transforming the evaluative logics through which access is converted into opportunity. Policies designed to redress inequality risk being partially neutralised when organisational actors continue to rely on culturally coded proxies for merit.

At the same time, South African firms operate within a regulatory environment that formally mandates demographic transformation, most notably through Broad‐Based Black Economic Empowerment (B‐BBEE) and employment equity requirements. These frameworks place pressure on organisations to recruit Black graduates and diversify their workforce. However, our findings suggest that such institutional mandates do not eliminate discretionary evaluation; rather, they reshape the conditions under which it operates. While organisations may comply with transformation targets at the level of recruitment pipelines, relatability continues to structure selection decisions within those pipelines. As a result, formal inclusion may coexist with informal filtering, where candidates who best approximate dominant cultural norms are preferred. This dynamic reveals a disjuncture between institutional compliance and organisational practice, in which regulatory frameworks address representation but leave intact the evaluative logics through which inequality is reproduced.

However, the empirical material also reveals a more complex dynamic than a purely unconscious account of bias would suggest. Many participants explicitly acknowledge the role of affinity, similarity, and subjective judgement in hiring decisions. Rather than denying these influences, they often frame them as necessary and organisationally rational, justified in terms of efficiency, risk management, and client expectations. This creates a tension in which evaluative practices are simultaneously recognised as partial and legitimised as pragmatic. In this sense, relatability does not operate solely as an implicit or hidden bias, but as a reflexively understood mechanism that is nonetheless normalised within organisational decision‐making. This helps explain how exclusion persists even in contexts where actors are aware of its dynamics, as the language of organisational necessity provides a moral and practical justification for reproducing inequality.

By linking micro‐level hiring practices to macro‐level structures of inequality, the study extends critical race theory's emphasis on the institutional embeddedness of racism (Delgado and Stefancic 2017). Although post‐apartheid labour markets are formally deracialised, spatial, educational, and network legacies continue to shape the distribution of cultural capital. Organisational cultures that valorise white middle‐class comportment translate these disparities into outcomes that appear meritocratic. The analysis also raises questions about the dominance of supply‐side policy interventions such as skills training, interview coaching, and employability programmes. These approaches leave demand‐side evaluative filters largely intact and risk reinforcing deficit narratives by encouraging excluded candidates to adapt to existing codes of professionalism without interrogating the codes themselves.

A more effective policy agenda would prioritise employer accountability through transparent hiring criteria, structured interview processes, and systematic auditing of recruitment outcomes. This could include requiring organisations to document and justify selection decisions, monitoring patterns of shortlisting and hiring across demographic groups, and embedding equity metrics within performance evaluation systems. In addition, reducing reliance on informal assessments of ‘fit’ requires the standardisation of evaluation criteria and the diversification of hiring panels to mitigate homophilous decision‐making. Broadening definitions of professionalism to encompass diverse styles, accents, and interactional registers requires sustained leadership commitment, the integration of equity metrics into performance evaluation, and the diversification of hiring panels. Isolated diversity training initiatives show limited durability. Durable change depends on reshaping the evaluative frameworks through which merit is recognised and legitimised. Without addressing these demand‐side evaluative filters, policy interventions risk improving access in form while reproducing exclusion in practice.

Recent initiatives in South Africa, such as Harambee Youth Employment Accelerator, have sought to disrupt conventional hiring practices by working with employers to redefine selection criteria, broaden access to opportunity, and reduce reliance on formal credentials and narrow definitions of fit (Maja and Ngcaweni 2023). These interventions demonstrate the potential for reshaping recruitment processes by making evaluation criteria more explicit, standardised, and inclusive. However, our findings suggest that such efforts must also address the deeper cultural and affective dimensions of hiring, including the implicit and reflexive reliance on relatability as a marker of organisational suitability.

This study introduces a conceptual vocabulary for analysing the persistence of racial inequality within ostensibly meritocratic labour markets. A striking feature of our data is that many of the hiring professionals responsible for these evaluations are themselves Black women or people of colour. This complicates any straightforward account of hiring as a process driven by racial bias alone. Rather than indicating the absence of inequality, this pattern points to the institutionalisation of evaluative norms that operate beyond individual racial identity. Hiring decisions are shaped within organisational fields structured by historically sedimented standards of professionalism, which are themselves products of classed educational pathways and institutional trajectories. As such, decision‐makers may reproduce these norms irrespective of their own racial positioning, drawing on shared understandings of competence, confidence, and client‐readiness that have been cultivated through similar institutional exposures. In this sense, relatability emerges as a racialised preference and a mechanism through which institutionally produced dispositions are recognised and valorised. This helps explain how racialised inequality persists even in contexts of increasing demographic diversity among organisational elites, as inclusion at the level of representation does not necessarily translate into transformation of evaluative frameworks.

On this basis, we extend racialised social systems theory by specifying how whiteness‐coded cultural capital functions as a gatekeeping device in recruitment. It brings Bourdieu's account of habitus into dialogue with theories of homophily and implicit bias to show how embodied performances are interpreted through longer histories of inequality. It also refines social closure theory by identifying relatability as a relational mechanism operating through embodied and affective dimensions of evaluation. Methodologically, the hermeneutic grounded approach combined interpretive depth with systematic theorisation, enabling the identification of tacit decision‐making logics that often escape conscious reflection. Reflexivity remained central, both in recognising researcher positionality and in acknowledging that naming relatability constitutes an intervention in debates over merit and equity. This highlights the importance of aligning institutional interventions with changes in everyday evaluative practices, rather than assuming that structural access alone will translate into equitable outcomes.

Future research could examine how relatability operates across intersecting axes of inequality, including gender, migration status, disability, and sexuality, as well as across labour markets structured by different racial orders. The expansion of AI‐mediated recruitment warrants particular scrutiny, as the incorporation of relatability cues into algorithmic systems risks amplifying exclusionary logics while rendering them less visible. Longitudinal research could further assess whether sustained organisational efforts succeed in diversifying the evaluative codes that define professionalism. By theorising relatability as a racialised construct, this article demonstrates how racial hierarchies are reproduced through ordinary hiring practices. It calls for a shift from interventions focused on individual employability toward reforms that address demand‐side evaluative processes. Without such a reorientation, the exclusion of Black African youth will continue to be legitimised through the language of comfort, readiness, and cultural proximity. Future research should examine how relatability functions in other segments of the labour market, including informal, low‐wage, and public‐sector employment contexts.

## Ethics Statement

This study received ethical clearance from the University of Cape Town (UCT) Faculty of Commerce Ethics Review Board. All participants provided informed consent prior to data collection. Participation was voluntary, and respondents were assured of confidentiality and the right to withdraw at any stage. Identifying details were anonymised to protect participant privacy. The study adhered to established ethical guidelines for research involving human participants.

## Conflicts of Interest

The authors declare no conflicts of interest.

## Data Availability

The data that support the findings of this study are available from the corresponding author upon reasonable request.
